# Contribution of Stretch-Induced Force Enhancement to Increased Performance in Maximal Voluntary and Submaximal Artificially Activated Stretch-Shortening Muscle Action

**DOI:** 10.3389/fphys.2020.592183

**Published:** 2020-11-12

**Authors:** Martin Groeber, Savvas Stafilidis, Wolfgang Seiberl, Arnold Baca

**Affiliations:** ^1^Department of Biomechanics, Kinesiology and Computer Science in Sport, Centre for Sport Science and University Sports, University of Vienna, Vienna, Austria; ^2^Department of Human Movement Science, Institute of Sport Science, Bundeswehr University Munich, Neubiberg, Germany

**Keywords:** force enhancement, force depression, elastic energy, electrical stimulation, muscular activation, concentric, eccentric

## Abstract

In everyday muscle action or exercises, a stretch-shortening cycle (SSC) is performed under different levels of intensity. Thereby, compared to a pure shortening contraction, the shortening phase in a SSC shows increased force, work, and power. One mechanism to explain this performance enhancement in the SSC shortening phase is, besides others, referred to the phenomenon of stretch-induced increase in muscle force (known as residual force enhancement; rFE). It is unclear to what extent the intensity of muscle action influences the contribution of rFE to the SSC performance enhancement. Therefore, we examined the knee torque, knee kinematics, m. vastus lateralis fascicle length, and pennation angle changes of 30 healthy adults during isometric, shortening (CON) and stretch-shortening (SSC) conditions of the quadriceps femoris. We conducted maximal voluntary contractions (MVC) and submaximal electrically stimulated contractions at 20%, 35%, and 50% of MVC. Isometric trials were performed at 20° knee flexion (straight leg: 0°), and dynamic trials followed dynamometer-driven ramp profiles of 80°–20° (CON) and 20°–80°–20° (SSC), at an angular velocity set to 60°/s. Joint mechanical work during shortening was significantly (*p* < 0.05) enhanced by up to 21% for all SSC conditions compared to pure CON contractions at the same intensity. Regarding the steady-state torque after the dynamic phase, we found significant torque depression for all submaximal SSCs compared to the isometric reference contractions. There was no difference in the steady-state torque after the shortening phases between CON and SSC conditions at all submaximal intensities, indicating no stretch-induced rFE that persisted throughout the shortening. In contrast, during MVC efforts, the steady-state torque after SSC was significantly less depressed compared to the steady-state torque after the CON condition (*p* = 0.034), without significant differences in the m. vastus lateralis fascicle length and pennation angle. From these results, we concluded that the contribution of the potential enhancing factors in SSCs of the m. quadriceps femoris is dependent on the contraction intensity and the type of activation.

## Introduction

A stretch-shortening cycle (SSC) is a muscle action that often occurs in everyday movements or sporting exercises. During a SSC, a lengthening contraction is immediately followed by a shortening contraction. This results in increased performance during the shortening phase compared to pure shortening contractions (“SSC-effect”) (Komi and Gollhofer, 1997; Komi, 2000). The mechanisms attributed to the enhanced force or work during the concentric phase of the SSC are the stretch-reflex (Dietz et al., 1979), the release of stored passive-elastic energy (Finni et al., 2001; Kawakami et al., 2002) and the pre-activation of muscles (Bobbert and Casius, 2005). The reflex motor response can enhance the ongoing contraction and thus stretch reflexes can make a net contribution to muscle stiffness in the SSC (Komi and Gollhofer, 1997). Tendinous tissue can store and recoil passive elastic energy, which can be utilized in a SSC (Finni et al., 2001). Pre-activation describes the time required for force development. In the SSC, the initial stretch allows muscles to build up force before shortening begins (van Schenau et al., 1997). An additional SSC mechanism that has been particularly under discussion in recent literature is related to stretch-induced force enhancing effects within the contractile element of muscles (Seiberl et al., 2015b; Hahn and Riedel, 2018; Tomolka et al., 2020).

It is well accepted that—compared to a length- and activation-matched reference contraction—an eccentric muscle action provides increased force or torque during, but also after, the lengthening phase, when the muscle is kept active in an isometric steady-state. The force or torque response to stretch is described to have two components. First, a velocity-dependent force enhancement (FE) throughout the stretch period (Edman, 2012). And second, the long-lasting component, which is known as the phenomenon of residual force enhancement (rFE) (Herzog, 2004). There is some experimental evidence that mechanisms related to stretch-induced rFE also contribute to enhanced performance in the SSC (Seiberl et al., 2015b; Fortuna et al., 2017; Fukutani et al., 2017a; Hahn and Riedel, 2018; Groeber et al., 2019). Different parameters influence the amount of rFE. For example, with increased stretch amplitude, the amount of rFE rises (Edman, 1978; Sugi and Tsuchiya, 1988), and it occurs at all muscle lengths (Rassier et al., 2003). On the other hand, no influence of stretch velocity could be detected for slow and moderate speeds (Edman et al., 1982; Lee and Herzog, 2002), whereas for fast stretch speeds, no significant rFE could be found (Fukutani et al., 2019a). These findings suggested rFE to be a muscle property which could be observed at different structural muscle levels (Abbott and Aubert, 1952; Edman, 1978; Herzog et al., 2003; Bullimore et al., 2007) and in different muscle groups (Oskouei and Herzog, 2006b; Pinniger and Cresswell, 2007; Seiberl et al., 2013). A very frequently used approach to explain rFE is the protein titin, which is thought to act as a kind of molecular spring. The binding of Ca^2+^ might enhance intramolecular attraction and compactness in the PEVK region of titin, which would be expected to increase titin stiffness (Rode et al., 2009; Herzog, 2014; Linke, 2018; Fukutani and Herzog, 2019). However, the exact role of titin is still unclear.

In contrast to an active stretch, the steady-state force or torque after a concentric contraction is reduced compared to an isometric reference contraction at the same muscle length (residual force depression, rFD) (Herzog, 2004). Thereby, an increase in shortening speed is associated with a decrease in rFD (Herzog and Leonard, 2000). In conformity with the force-velocity relationship (Hill, 1938), increased contraction velocities lead to reduced force capacities resulting in reduced work produced during shortening. Thus, the amount of rFD increases with the amount of work during shortening. Accordingly, rFD declines with decreasing shortening magnitude (Maréchal and Plaghki, 1979; Herzog and Leonard, 1997). Stress-induced inhibition of the actin-myosin overlap zone is the primary mechanism suggested for rFD (Herzog, 2004; Joumaa et al., 2017). In addition, the protein titin might bind to actin upon muscle activation. This could lead to an inhibition of cross bridges by less binding sites for myosin on the actin filament due to bound titin (Rode et al., 2009).

These often independently researched phenomena of rFD and rFE are directly confronted in stretch-shortening cycles. If analyzed together in the context of SSCs, conflicting results for the interaction of these force-enhancing and -depressing history-dependent properties are presented in literature. For example, Seiberl et al. (2015b) and Fortuna et al. (2017) reported a contribution of rFE to the SSC performance enhancement by counteracting the development of rFD which is established during the concentric phase. In contrast, Herzog and Leonard (2000) and Lee et al. (2001) reported the same amount of rFD after a SSC as for pure concentric muscle actions. Thus, possible interactions of rFE- and rFD-related mechanisms in SSC, thereby influencing muscle performance, are still not well understood.

The standardized protocols for evaluating the history-dependent properties of muscle action may not adequately represent everyday movements like walking or running, since the operating conditions vary during muscle action with these natural movements (Bohm et al., 2018). However, in some resistance training exercises the magnitude and velocity of the eccentric and concentric phase is kept constant, especially when using lower intensities (Sakamoto and Sinclair, 2006, 2012). Although these parameters are similar in the eccentric and concentric phase, the entire SSC is often performed under different levels of intensity in resistance training exercises in order to regulate training exposure (Wernbom et al., 2007).

Concerning above mentioned mechanisms related to the performance enhancement in SSCs, it can be assumed, that the intensity of muscle action differently affects their contributions to increased force, work or power. For example, it is reported that rFE increases with increasing contraction intensity (Oskouei and Herzog, 2006a). However, this result could not be confirmed in a feedback-controlled submaximal knee extension protocol study (Seiberl et al., 2012). Owing to that, if contraction intensity could influence rFE, it would also influence performance in a SSC. Also, if the proposed mechanism of titin—which spans from the Z line to the M band of the sarcomere (Leonard and Herzog, 2010)—contributes to the enhancing effects, elastic titin forces might increase upon higher muscle activation (Fukutani and Herzog, 2019), meaning elevated Ca^2+^ ion concentration in the environment of the myofibrils (Gehlert et al., 2015). Additionally, tendon elongation should be greater with increasing muscle force (Wang, 2006; Fukutani et al., 2017b), resulting in more elastic energy stored in the tendon during the eccentric phase, which is beneficial in the shortening phase of the SSC, but should not account solely for enhanced performance in a SSC (Seiberl et al., 2015b). Hence, the focus of this work was to examine the influence of contraction intensity on the history-dependent properties of muscle action in a SSC.

We hypothesized that with increasing intensity the SSC-effects are larger; possibly due to increased rFE, which should be observable by enhanced torque compared to a pure concentric (CON) muscle action in the steady-state after the SSC.

## Materials and Methods

### Participants

Thirty healthy adults initially participated in this study. Three subjects did not complete the tests, or data was missing; therefore, they were excluded from further analysis. Finally, data were obtained from 12 female and 15 male adults (age: 26.6 ± 6.2 years, height: 175.4 ± 8.7 cm, body mass: 71.8 ± 11.3 kg), they all participated voluntarily and provided written informed consent prior to the study. The suitability of participation was determined with an anamnesis questionnaire to determine the risk factors for physical activity. The participants had no neuromuscular disorders, cardiovascular problems or injury to the right leg. The Ethics Committee of the University of Vienna approved the experimental protocol (reference number: 00364).

### Experimental Setup

Knee joint torque was measured using an isokinetic dynamometer (HUMAC Norm, Model 770; CSMi) at the right leg. We captured all analog signals of the isokinetic dynamometer with the Vicon Nexus A/D card (16 bit) with a sampling frequency of 2 kHz. The upper body was fixed to the dynamometer by straps (100° seat back angle). The sitting position was precisely adapted to each test person. The lateral femoral condyles of every participant were aligned with the rotation axis of the dynamometer.

Synchronously, kinematic data was recorded with a Vicon Nexus motion-capturing system (Oxford, United Kingdom, 100 Hz) using nine cameras (Vantage V8). Eight reflective markers were captured, fixed at the following positions: trochanter major, the most prominent points of the medial and lateral femoral condyles, medial and lateral malleolus of the right leg. Additionally, we placed one marker at the axis of the dynamometer and two markers on the dynamometer’s arm (one of them at the point of force application defining the lever arm as the distance between the line of action of the applied extension force and the dynamometer’s axis of rotation).

For all voluntary contractions, the EMG signals of the m. vastus medialis and m. rectus femoris were captured (Delsys, Trigno Wireless EMG System, United States, 2 kHz) and recorded synchronously with the torque and kinematic data. The electrodes were attached following the guidelines of the SENIAM group (Hermens et al., 2000). The sensors had an inter-electrode spacing of 10 mm (Delsys, Trigno Avanti Sensor).

Muscle contractions were evoked at specific intensities by electrical stimulation (Digitimer DS8R, United Kingdom). The areas of the skin were shaved and cleaned with alcohol for the later application of the electrodes. The muscle motor points of m. vastus lateralis and m. vastus medialis were identified by scanning the skin’s surface with a motor point pen (COMPEX, United Kingdom). Two electrodes (5 × 5 cm) were placed precisely on the previously identified motor points (Gobbo et al., 2014). Repetitive 100 μs square-wave pulses at 100 Hz were used. The train duration was approximately 6 s long.

**FIGURE 1:**
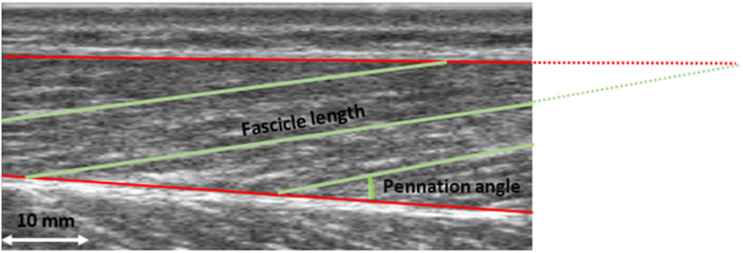
Ultrasound image of the m. vastus lateralis. Fascicle length and pennation angle were determined for three fascicles. Pennation angle was calculated between the muscle fascicle and the deep aponeurosis. Fascicle length was defined as the distance between the intersection of the upper aponeurosis with the muscle fascicle and the intersection of the lower aponeurosis and muscle fascicle. The mean of the three measured values was used for further analysis.

For the investigation of history dependent effects in muscle action it is crucial that experimental conditions concerning muscle activity and muscle length are well controlled. Especially muscle length not necessarily follows dynamometer angular settings in a linear manner for different conditions and intensities, what can bias the interpretation of force capacities according to the force-length relationship of the knee extensors. For this reason, we additionally used ultrasonography (Telemed ArtUS EXT-1H, IT, 70 Hz) to determine the underlying fascicle length changes and pennation angle of the m. vastus lateralis (see Figure 1). A linear probe (LV8-5N60-A2) with a field of view of 60 mm was used. The probe was fixed to the muscle belly of the m. vastus lateralis with a custom-made bracket. To synchronize the video data with the other data recorded with Vicon Nexus, an analog signal (0–3 V) was generated when the video was started and stopped. This analog signal was captured by the Vicon system.

### Experimental Protocol

**FIGURE 2:**
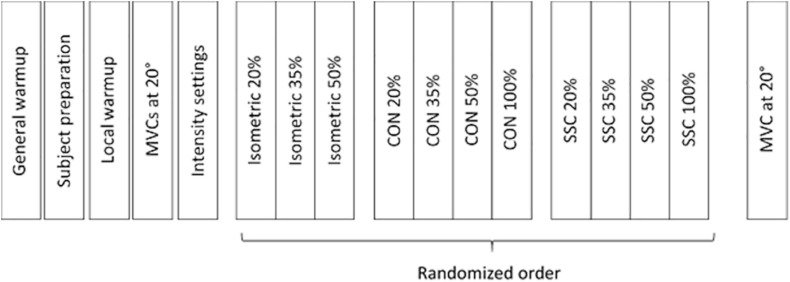
Experimental protocol work flow. The test protocol comprised maximal voluntary contractions (100%) and submaximal electrical stimulated contractions at 20%, 35%, and 50% of MVC.

On test day, the participants first had a general warm-up (bicycle ergometer, 5 min, between 80 and 90 W), which was followed by a specific warm-up of the quadriceps femoris on the isokinetic dynamometer (several submaximal contractions, 5 min). After the warm-up, the subjects had to perform two maximal voluntary contractions (MVCs) at a 20° dynamometer angle (full knee extension was defined as 0°). There was no statistical difference (*t*-test paired, *p* = 0.089) between the two MVC contractions (3.8% on average) and the average of peak joint torque of both trials was defined as 100% intensity. As a next step, current was applied and incrementally increased until the desired isometric knee torque was reached (20, 35, and 50% of MVC). Once the desired intensity was reached, the stimulus was maintained for several seconds to ensure a steady torque response. These current intensity settings were used for the entire experiment. The settings were checked again after a pause of one minute. The protocol comprised isometric contraction (ISO), pure shortening contractions (CON) and stretch-shortening cycles (SSC). It should be noted that the term ‘isometric’ is used for simplicity, although it actually refers to a fixed-end muscle-tendon unit contraction, where some muscle shortening at initial activation is likely, even when the joint angle is constant (Fukashiro et al., 1995). The angular velocity was fixed at 60°/s, and a fixed range of motion adjusted by the dynamometer (ISO: 20°, CON: 80°–20°, SSC: 20°–80°–20°) was used. This range of motion refers to the ascending limb of the knee extensor torque-angle relationship (Hahn et al., 2007) and reflects SSC ranges as found in many everyday movements such as walking, running or hopping (Jin and Hahn, 2019). In randomized order, MVC and different submaximal contraction intensities were tested (20, 35, and 50% of MVC) that were triggered through electrical stimulation. For simplified designation, tests under maximal voluntary contraction will be referred to as 100% (Figure 2). Rest between the contractions was two minutes in order to prevent fatigue (Tilp et al., 2011).

Dynamic contractions had an isometric pre-activation period before the dynamic phase (until a plateau was reached) followed by an isometric hold phase (steady-state) after the knee rotation. At the end of the experimental protocol, the subjects had to perform one fixed-end MVC at 20° dynamometer angle.

### Data Processing

**FIGURE 3:**
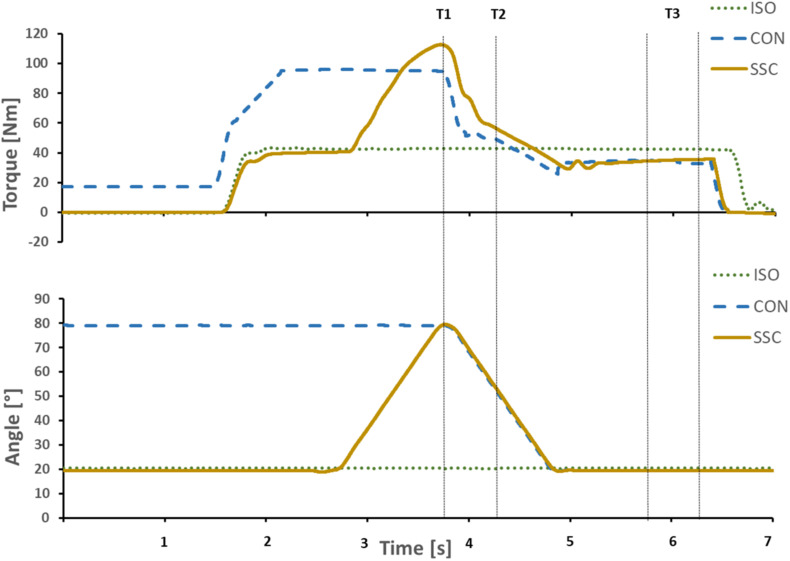
Exemplary representation of torque-time and angle-time traces. Vertical lines indicate the time point at the end of stretch (T1), midpoint of shortening (T2), and the steady-state interval where the mean steady-state torque was calculated (T3). Continuous yellow line SSC, dotted green line ISO, and dashed blue line CON.

Torque and dynamometer angle data were low-pass filtered (zero-delay, fourth-order Butterworth) with a cut-off frequency of 10 Hz. Gravitational forces acting on the dynamometer arm system were corrected for all subjects. Each contraction was repeated twice and mean values were used for further analysis. CON and SSC were compared at T1 (80° dynamometer angle, end of stretch for SSC condition) and at 50° dynamometer angle (midpoint of 80°–20° range, T2) in the shortening phase. ISO, CON, and SSC were compared at the isometric steady-state, 1–1.5 s after the dynamic phase (T3, 20° dynamometer angle), while at that point the average values of 0.5 s were used (Figure 3).

Additionally, the mechanical work during angular rotation was calculated as the integral of torque during the shortening phase, using a numerical trapezoidal method.

(1) W=∫M⁢d⁢φ=∫M⋅ω⁢dt

with *W* as mechanical work [J], *M* as torque [Nm], and ω as rotational velocity [rad/s].

Due to difficulties in the standardization of the knee angle range of motion at high torque levels, mechanical work values were additionally adjusted to the respective range of motion in each individual trial (see results section). Different knee angles between CON and SSC at T1 affect the results of the absolute mechanical work during the shortening. Therefore, we decided to adjust mechanical work to the respective range of motion to avoid this problem.

Kinematic data was also low-pass filtered using a cutoff frequency of 10 Hz. Knee-joint angle was compared between conditions at time points T1, T2, and T3. It had been previously shown that due to the compliance of the dynamometer and the tissue deformation, a shift of both knee joint and dynamometer axis occurs, and therefore differences between the measured and the resultant knee joint moments exist. To address this shortcoming, we implemented the inverse dynamic approach proposed by (Arampatzis et al., 2004).

(2) $$M_{\mathrm{res}}=M_{\mathrm{Meas}}\cdot \frac{d_{K}}{d_{B}}$$

with ***M*_*res*_** as the corrected joint moment, ***M*_*Meas*_** the initially measured moment, ***d*_*B*_** as the lever arm of the applied force to the dynamometer axis and ***d*_*K*_** as the lever arm of force to the knee joint [according to the free body diagram reported by Arampatzis et al. (2004)].

EMG data were band-pass filtered (10–400 Hz, fourth-order Butterworth), rectified, smoothed (250 ms moving average). Mean difference in m. rectus femoris and m. vastus medialis activity was used for analysis.

Pennation angle was calculated between the muscle fascicle and the deep aponeurosis. Fascicle length was defined as the distance between the intersection of the superficial aponeurosis with the muscle fascicle and the intersection of the deep aponeurosis and muscle fascicle. This was always done for three fascicles and the mean value was calculated (see Figure 1). If the muscle fascicle was no longer visible on the image section, the point of intersection was calculated by assuming a linear continuation. Trigonometry was used to estimate the part of the fascicle that was not visible. This approach has been widely used before; nevertheless, it should be noted that this linear approach could result in an error. However, it was reported that this error is less than 2.4% (Narici et al., 2003; Reeves and Narici, 2003). Two evaluators digitized the ultrasound image separately for testing on interrater reliability, using open-source software (Tracker, Open Source Physics, Version 5.1.1).

The synchronization of all the data was achieved by use of the ultrasound device’s start-stop analog signal.

### Statistical Analysis

Data was tested for normality using a Kolmogorov–Smirnov test. Depending on the number of conditions to be compared, either a paired t-test with dependent variables or repeated measures ANOVA was employed. If sphericity was violated, Greenhouse-Geisser correction was used. Two-way ANOVA (within-within subject design) was adopted to examine the interaction (condition × intensity) and main effect (condition and intensity) for knee joint torque, mechanical work, knee angle, fascicle length and pennation angle. If the interaction (condition × intensity) was significant, subsequent *post hoc* comparisons with Bonferroni adjustments were conducted for comparing CON and SSC at each level of intensity. One-way ANOVA or paired t-test was utilized for EMG. The level of significance was set to *p* < 0.05. The effect size was assessed with partial eta squared (η^2^). Interrater reliability for pennation angle and fascicle length measurements was analyzed by calculating the intraclass correlation coefficient (ICC, two-way mixed model; single measures).

By means of a paired *t*-test, the MVC at the end of the test session was compared with the MVC at the beginning to determine possible fatigue. For comparison of ISO, CON, and SSC conditions, the previously described time points (T1–T3) were used. Data are presented as mean ± SD.

## Results

### Initial Conditions

The achieved isometric (ISO) joint torque at the specified percentages (20, 35, and 50% of MVC) reached a mean value of 23.2 ± 6.0%, 36.2 ± 8.0%, and 49.3 ± 9.4%, respectively, at the same dynamometer angle of 20°.

Further, a *t*-test revealed no statistical difference between MVCs at the beginning (92.5 ± 29.9 Nm) and at the end of the test protocol (91.3 ± 29.2 Nm), indicating no fatigue.

### Joint Torque and Work Measurements

**FIGURE 4:**
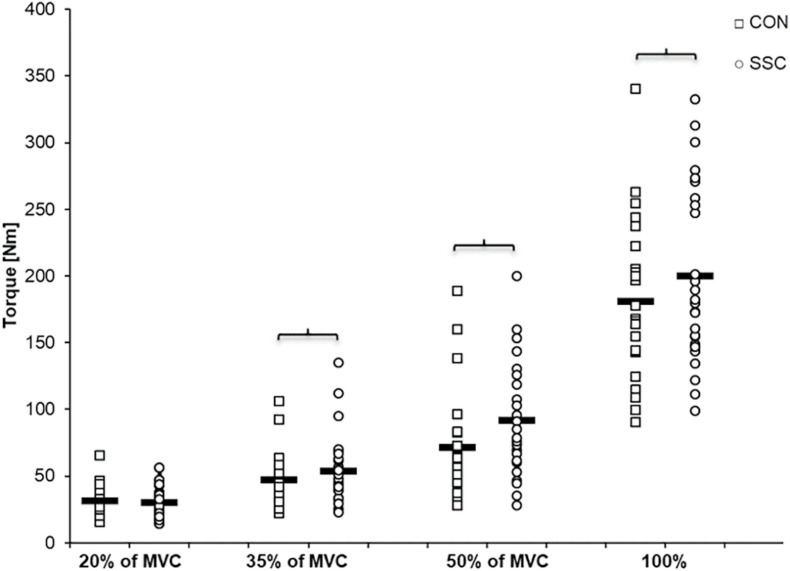
Mean (*n* = 27, thick horizontal line) (and mean of each individual subject) values of joint torque at the onset of shortening (T1). Squares represent concentric condition (CON), whereas circles represent stretch-shortening conditions (SSC). Braces indicate significant (*p* < 0.05) differences between CON and SSC at the same intensity level.

Two-way ANOVA revealed a significant interaction (condition × intensity) of torque after the stretch phase (T1) (*p* = 0.023, η^2^ = 0.159). Main effect of intensity (*p* < 0.001, η^2^ = 0.843) and for condition (*p* < 0.001, η^2^ = 0.501) revealed increased torque with higher intensity and for the SSC condition. For the intensities of 35, 50, and 100%, the torque after the stretch phase (T1) in SSCs was significantly higher compared to the isometric pre-activation in the CON conditions at the same activation level and the corresponding dynamometer angle (35%: *p* = 0.013; 50%: *p* = 0.001, and 100%: *p* = 0.032). This resulted in 13.7, 32.2, and 10.7% FE in the 35, 50, and 100% intensity conditions. No FE could be found for the lowest (20%) activation level (*p* = 0.103) (see Figure 4 and Table 1).

**TABLE 1:** Mean (±SD; *n* = 27) values of knee joint torque at T1 (onset of shortening) and T3 (steady-state after dynamic phase).

Contraction intensity	Knee joint torque [Nm ± SD]				
	
	T1		T3		
		
	CON	SSC	ISO	CON	SSC
20%	31.1 ± 10.7	29.8 ± 10.3	18.2 ± 4.8	17.1 ± 4.5	16.7 ± 3.6^∗^
35%	**47.2 ± 19.6**	**53.7 ± 25.8**	29.9 ± 6.9	26.6 ± 7.0^∗^	25.7 ± 6.1^∗^
50%	**71.6 ± 37.8**	**94.7 ± 50.3**	40.4 ± 9.6	35.3 ± 10.7^∗^	34.0 ± 10.1^∗^
100%	**180.7 ± 62.1**	**200.0 ± 66.4**	76.4 ± 23.5	**67.4 ± 22.2**^∗^	**74.1 ± 25.1**

*Bold values indicate significant (*p* < 0.05) differences between the shortening (CON) and stretch-shortening (SSC) condition at the respective intensity level and time-point. Asterisks indicate a significant difference of CON or SSC to the isometric reference contraction (ISO) at the respective intensity level and time-point.*

**FIGURE 5:**
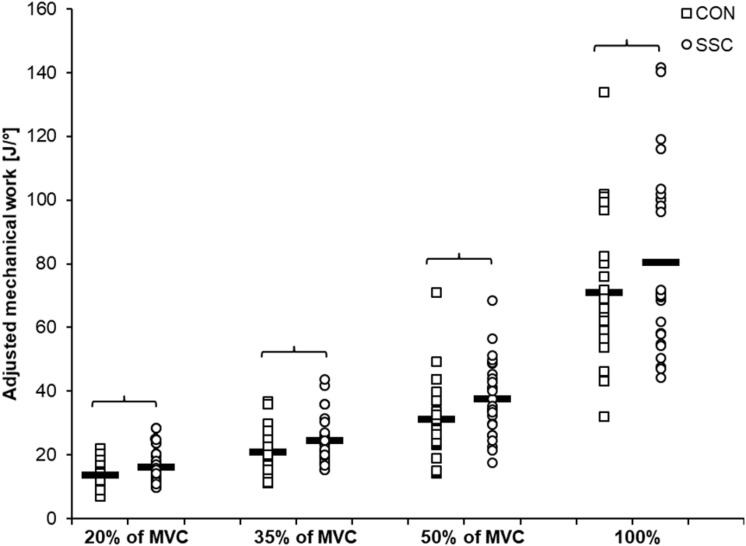
Mean (*n* = 27, thick horizontal line) (and mean of each individual subjects) values of mechanical work adjusted to the actual knee rotation during the shortening phase. Squares represent concentric condition (CON), whereas circles represent stretch-shortening conditions (SSC). Braces indicate significant (*p* < 0.05) differences between CON and SSC at the same intensity level.

Significant interaction (condition × intensity) was found for the range-adjusted mechanical work (*p* = 0.049, η^2^ = 0.123). Main effect of intensity (*p* < 0.001, η^2^ = 0.839) and condition (*p* < 0.001, η^2^ = 0.708) revealed increased mechanical work with higher intensity and for the SSC condition. Range-adjusted mechanical work during shortening was significantly higher for all SSCs compared to CON contractions at the same activation level (20%: *p* < 0.001; 35%: *p* < 0.001; 50%: *p* < 0.001; 100%: *p* = 0.001) (see Figure 5 and Table 2). The absolute mechanical work during shortening was also significantly higher for all SSC conditions compared to the CON conditions at the same intensity (*p* < 0.05). The percentage increase of normalized mechanical work in the SSC compared to CON contraction (referred to as SSC-effect) was 17.4, 17.7, 20.9, and 13.1% for the 20, 35, 50, and 100% intensities, respectively.

**TABLE 2:** Mean (±SD; *n* = 27) values of the absolute mechanical work and the work adjusted to the actual knee rotation during shortening.

Contraction intensity	Absolute mechanical		Adjusted mechanical	
		
	work [J ± SD]		work [J/° ± SD]	
		
	CON	SSC	CON	SSC
20%	**763.8 ± 184.2**	**869.8 ± 182.2**	**13.8 ± 3.5**	**16.2 ± 4.4**
35%	**1150.0 ± 321.6**	**1295.9 ± 365.3**	**20.9 ± 6.9**	**24.6 ± 7.6**
50%	**1648.1 ± 525.5**	**1911.6 ± 558.0**	**31.1 ± 11.9**	**37.6 ± 11.7**
100%	**3458.9 ± 1044.9**	**3733.6 ± 1182.6**	**71.1 ± 24.0**	**80.4 ± 28.6**

*Bold values indicate significant (*p* < 0.05) differences between the shortening (CON) and stretch-shortening (SSC) condition at the respective intensity.*

**FIGURE 6:**
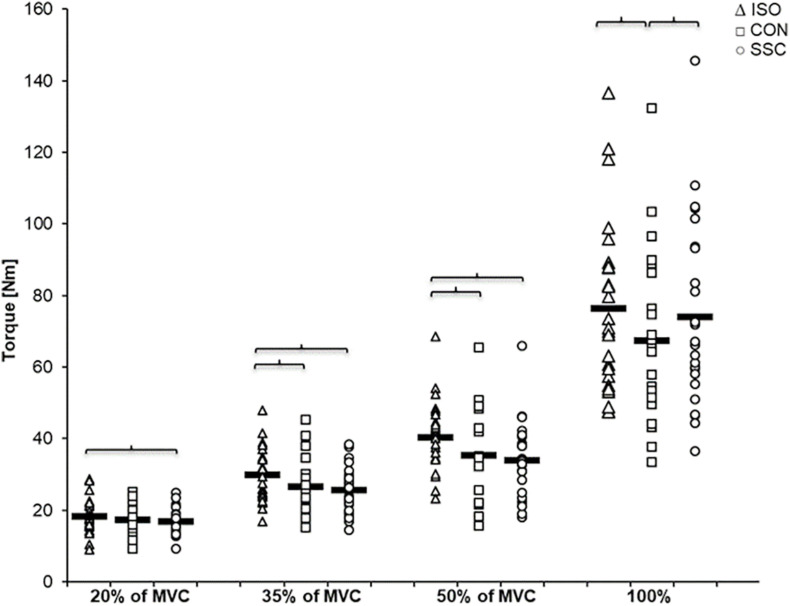
Mean (*n* = 27, thick horizonal line) (and mean of each individual subject) values of joint torque in the steady-state after the dynamic phase (T3). Triangles represent purely isometric torque at 20° knee flexion (ISO), squares represent concentric condition (CON), whereas circles represent stretch-shortening conditions (SSC). Braces indicate significant (*p* < 0.05) differences between the conditions.

Regarding the steady-state torque after the dynamic phase (T3), two-way ANOVA with repeated measures revealed a significant interaction (*p* = 0.004, η^2^ = 0.207). Main effect of intensity (*p* < 0.001, η^2^ = 0.856) and condition (*p* < 0.001, η^2^ = 0.530) was significant. *Post hoc* comparisons revealed significant rFD for all SSCs compared to the isometric reference conditions for all submaximal levels of contractions (20–50%). A significant torque depression of the CON contractions (CON_35%: 11.1%, CON_50%: 12.7%, CON_100%: 11.8%) could be found for all activation levels except 20% of MVC (CON_20%: 6.6%, no significance, *p* = 0.288). The only difference between CON and SSC was found under 100% (*p* = 0.034), where the SSC steady-state torque was significantly less depressed than in the CON condition (see Figure 6 and Table 1).

### Knee Angle

**FIGURE 7:**
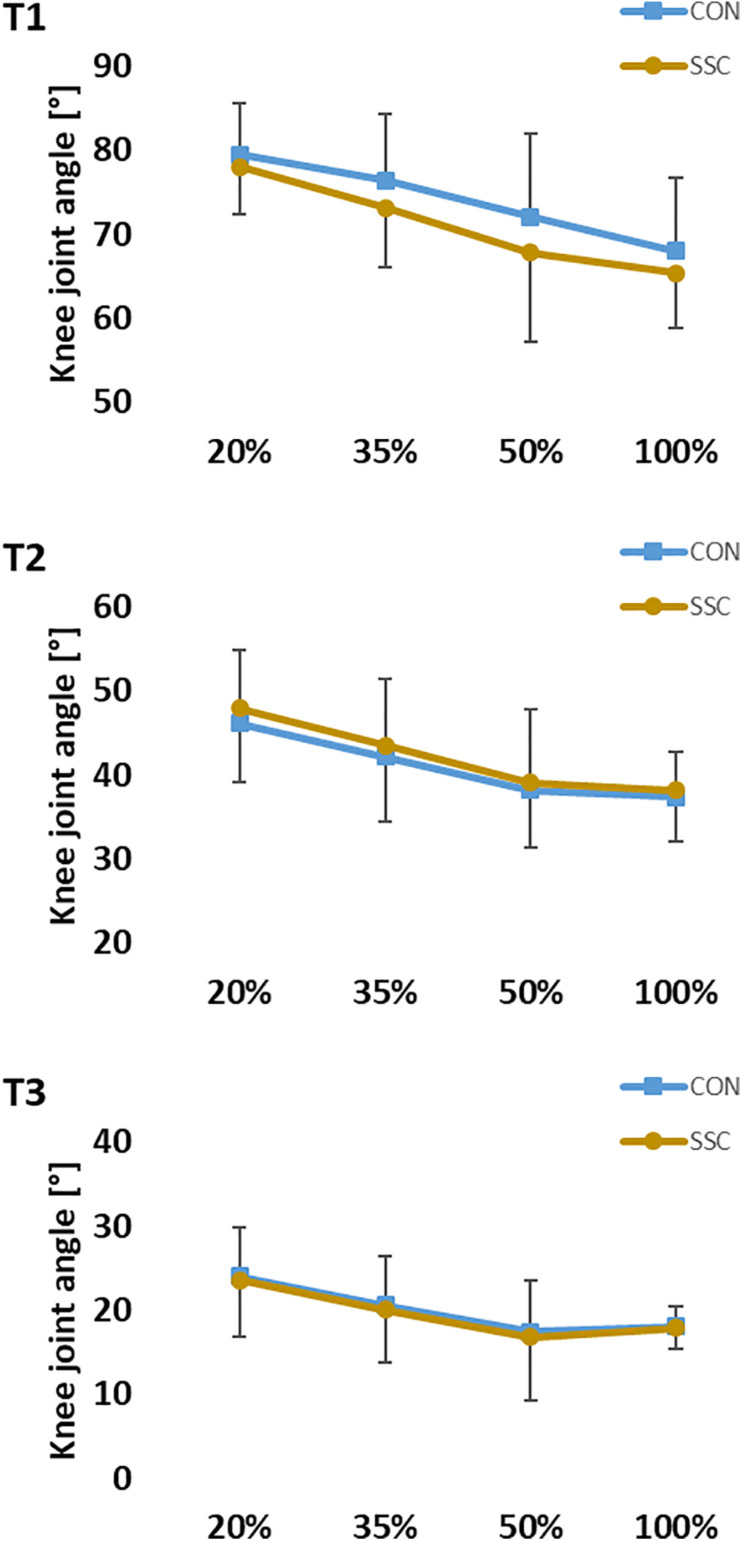
Mean (±SD, *n* = 27) values of knee joint angle at different contraction intensities (% of MVC). T1 is the time-point at the onset of shortening, T2 in the middle of the shortening phase, T3 the time-point at steady-state after the dynamic phase. No significant interaction (condition × intensity) was found. Main effect of condition revealed, that knee joint flexion angle was significantly (*p* < 0.05) higher at the CON compared to the SSC condition at T1.

For knee angle, no significant interaction (condition × intensity) was found at all time points (T1: *p* = 0.251, η^2^ = 0.052; T2: *p* = 0.309, η^2^ = 0.015; T3: *p* = 0.736, η^2^ = 0.012). Main effect of intensity was found at all three time points (T1: *p* < 0.001, η^2^ = 0.800; T2: *p* < 0.001, η^2^ = 0.648; T3: *p* < 0.001, η^2^ = 0.412), knee joint flexion angle decreased significantly as the intensity raised. The main effect of condition was only significant at T1 (*p* < 0.001, η^2^ = 0.557), knee joint flexion angle was significantly higher at the CON condition compared to the SSC condition (see Figure 7).

### Muscle Architectural Changes

Analysis of the fascicle length and pennation angle was obtained by two independent investigators. Mean ICC across all measurements was 0.84 (ranging from 0.80 to 0.87 for subjects) for fascicle length and 0.81 (ranging 0.77–0.85 for subjects) for pennation angle, indicating good interrater reliability (Koo and Li, 2016).

**FIGURE 8:**
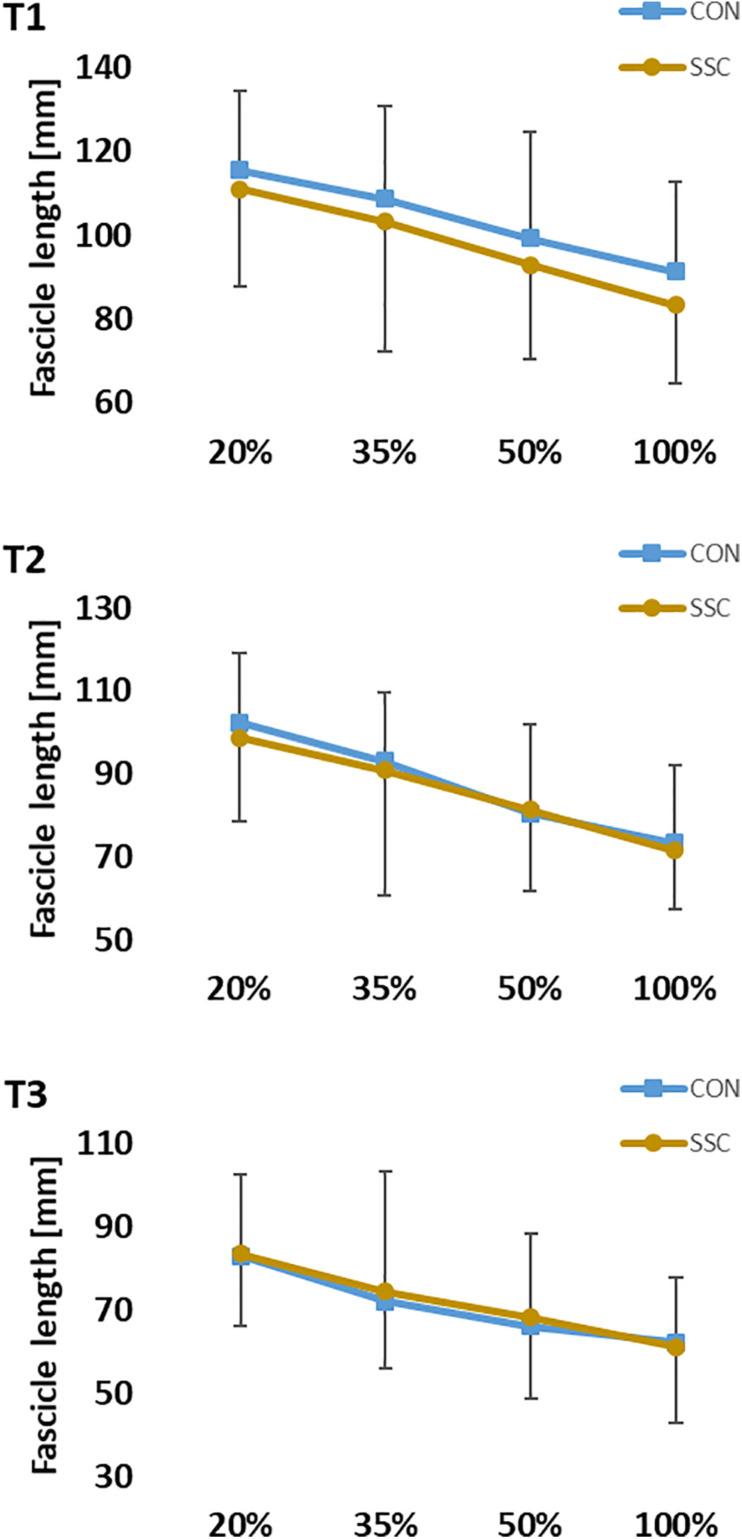
Mean (±SD, *n* = 27) values of fascicle length at different contraction intensities (% of MVC). T1 is the time-point at the onset of shortening, T2 in the middle of the shortening phase, T3 the time-point at steady-state after the dynamic phase. No significant interaction (condition × intensity) was found. Main effect of condition was also not significant (*p* > 0.05).

The interaction (condition × intensity) was not significant for fascicle length at all time points (T1: *p* = 0.667, η^2^ = 0.015; T2: *p* = 0.638, η^2^ = 0.021; T3: *p* = 0.717, η^2^ = 0.021). Main effect of intensity showed significant shorter fascicle length with increased intensity (T1: *p* < 0.001, η^2^ = 0.582; T2: *p* < 0.001, η^2^ = 0.588; T3: *p* < 0.001, η^2^ = 0.456). Main effect of condition was not significant at all time points (T1: *p* = 0.068, η^2^ = 0.150; T2: *p* = 0.413, η^2^ = 0.032; T3: *p* = 0.257, η^2^ = 0.061) (see Figure 8).

**FIGURE 9:**
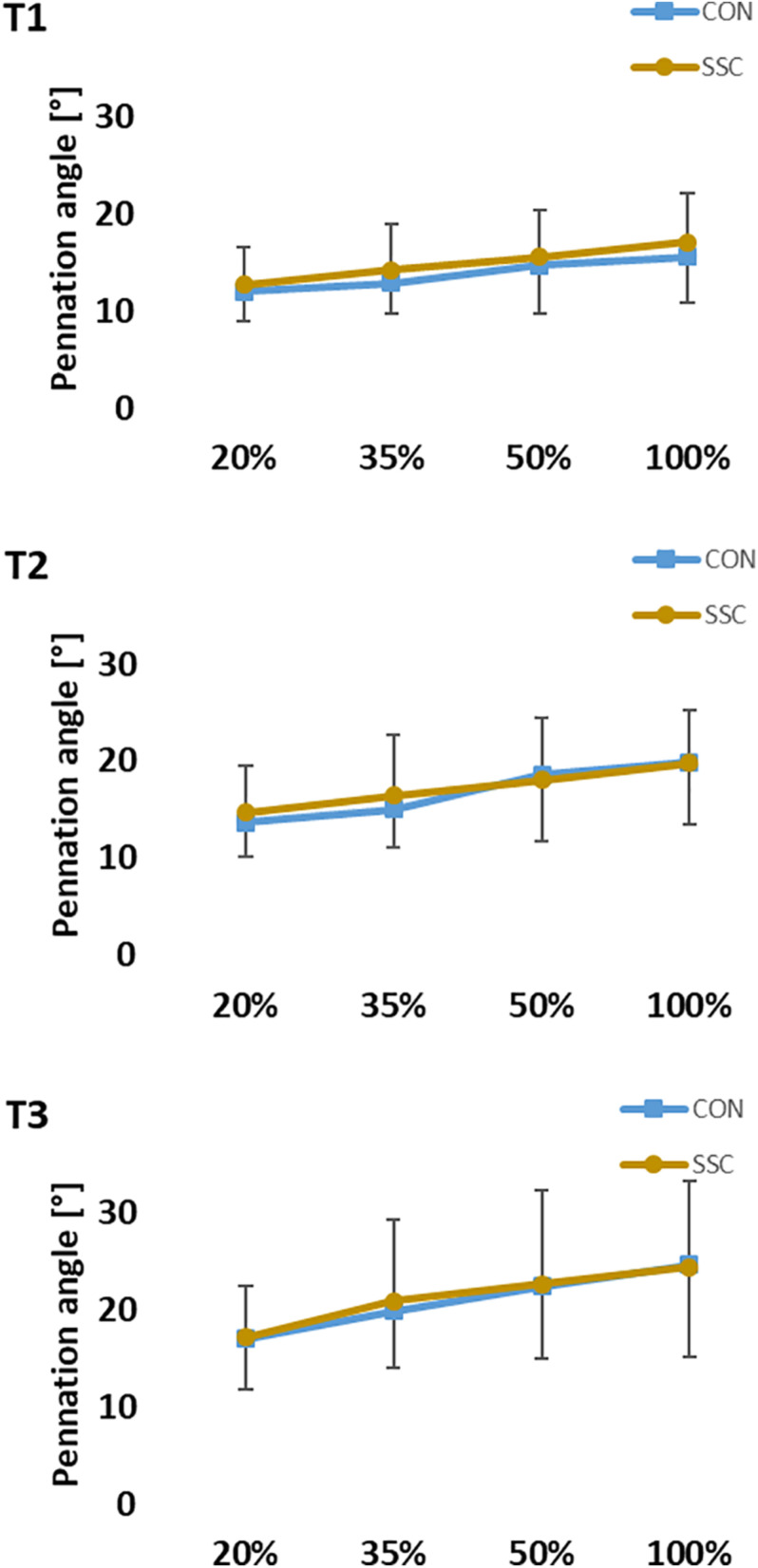
Mean (±SD, *n* = 27) values of pennation angle at different contraction intensities (% of MVC). T1 is the time-point at the onset of shortening, T2 in the middle of the shortening phase, T3 the time-point at steady-state after the dynamic phase. No significant interaction (condition × intensity) was found. Main effect of condition revealed, that the pennation angle was significantly (*p* < 0.05) higher at the SSC compared to the CON condition at T1.

Two-way ANOVA revealed no significant interaction (condition × intensity) of pennation angle at all time points (T1: *p* = 0.620, η^2^ = 0.021; T2: *p* = 0.232, η^2^ = 0.067; T3: *p* = 0.742, η^2^ = 0.015). Whereas the main effect of intensity showed, that pennation angle was increased with higher intensity (T1: *p* < 0.001, η^2^ = 0.550; T2: *p* < 0.001, η^2^ = 0.630; T3: *p* < 0.001, η^2^ = 0.452). Pennation angle was significant higher in the CON condition compared to the SSC at T1. No statistical difference could be found at T2 and T3 (T1: *p* = 0.023, η^2^ = 0.224; T2: *p* = 0.392, η^2^ = 0.035; T3: *p* = 0.984, η^2^ < 0.001) (see Figure 9).

### Muscle Activity

For the comparison of SSC and CON muscle activity, we did not find any differences for all analyzed muscles and time points (*p* > 0.05). Between SSC and CON conditions the mean difference in m. rectus femoris activity was 3.1 ± 4.8%, 5.5 ± 5.6%, and 4.3 ± 4.0% at T1, T2, and T3, respectively. For m. vastus medialis, the mean difference between SSC and CON activity at T1, T2, and T3 was 8.6 ± 9.8%, 6.1 ± 5.0%, and 2.6 ± 3.4%.

At T3, in the steady-state phases after shortening, SSC and CON muscle activity was additionally compared to isometric references. Here, m. rectus femoris also did not show any statistical difference between conditions. For m. vastus medialis, compared to the ISO condition we found significantly lower muscle activity for both, the CON (22.5 ± 30.9%) and the SSC (20.6 ± 30.3%) condition (*p* < 0.001, η^2^ = 0.36).

## Discussion

The study was designed to investigate the influence of the contraction intensity on stretch-induced performance enhancement in a SSC. We hypothesized that the SSC-effects (enhanced work during shortening phase) are larger with increasing intensity, partly explainable by increased contributions of stretch-induced mechanisms related to rFE. As expected, we found that the average work values were significantly greater for all SSCs compared to the corresponding concentric contractions. The SSC-effect was about 20% in the submaximal electrically stimulated trials, and about 13% in MVCs. Interestingly and against our expectations, despite the clear performance enhancement during SSCs, all shortening phases ended in significant rFD, except for SSCs performed at 100% intensity.

Similar to other studies, we found increased joint torque at the end of stretch compared to the isometric pre-activation in the CON condition (Power et al., 2013; Seiberl et al., 2015b; Hahn and Riedel, 2018), but not for all intensity levels. We did not see a FE at the end of stretches with the lowest activation (20% of MVC), but increasing FE from 13 to 32% with increasing electrical stimulation (Figure 4). Thus, our results indicate that the FE during the stretch phase is intensity dependent—at least during electrical stimulation. During voluntarily maximally activated SSCs, the torque was also significantly enhanced compared with isometric pre-activation of the CON condition; but this FE of about 11% at 100% intensity was slightly lower than the FE at 35% intensity in the electrically stimulated trials. Similar to our study, Lee and Herzog (2002) compared peak forces during eccentric contractions in m. adductor pollicis and also found that slightly less FE was produced under MVC conditions compared to electrically stimulated contractions at an intensity the subjects could comfortably tolerate. A lack of increased force in voluntary eccentric contractions is typically associated with an inhibition of the neural drive (Westing et al., 1990; Webber and Kriellaars, 1997), which could explain the lower relative FE at MVCs compared with the submaximally electrically stimulated trials (≥35%). However, examination of the EMG data at the end of the stretch phase in SSCs showed no significant differences (*p* > 0.05) to the CON condition at the respective time point in the isometric pre-activation phase before shortening, which further means that also an inhibition of neural drive cannot explain these results. Our results indicate that relative FE increases with higher submaximal intensities. At the MVC level, the relative FE is smaller than during the electrically stimulated attempts (>35%).

The kinematic analysis revealed that there was a discrepancy between dynamometer-defined and measured knee angle. In the CON condition, the knee joint angle was significantly more flexed compared to the SSC condition. Although we did not find any statistical interaction (condition × intensity), the difference in knee joint angle between the conditions had the tendency to get bigger with increasing intensity. Accordingly, the highest discrepancy was found at the end of the stretch (T1) during MVC stretch-shortening trials, where the highest absolute torques were measured. Although the subjects were firmly strapped to the seat of the dynamometer, the high torques during the eccentric phases can lead to a slight elevation of the pelvis, what in turn gives way for the thigh and counteracts exact dynamometer-driven knee flexion. The significantly lower knee joint angles (more extended) at T1 led to architectural differences in pennation angle of m. vastus lateralis in these trials. Although we found no statistical difference in fascicle length (Figure 8), it can be assumed that the differences in knee joint and pennation angle influenced force and torque production according to the force-length and torque-angle relationship of knee extensors. As during this experiment the knee extensors likely worked on the ascending limb of the torque-angle relationship (Marginson and Eston, 2001; Hahn et al., 2007), the standardization difficulties possibly lead to an underestimation of the relative force enhancement at the end of stretch. We assume that a perfectly matched eccentric-concentric turning-point at a greater knee flexion angle would have generated even higher FE. This is also supported by data from the m. vastus lateralis confirming that the presented fascicle lengths in this work can be related to the ascending limb of the respective force-length relationship (Nikolaidou et al., 2017). Therefore, we believe it is safe to assume that there was clear FE in all SSC conditions, except for at the lowest intensities. However, since we did not assess the force-length relationship of each individual subject, the interpretation of an underestimated FE in these cases remains speculative. As already mentioned before, the range of motion was deliberately chosen, since it reflects SSC ranges as found in many everyday movements (Jin and Hahn, 2019). However, it should be noted that the results could be also different dependent on where the muscle operates on the force-length relationship. Under well controlled conditions *in vitro* or *in situ* studies, eccentric force enhancement during and after stretch is typically greater at longer sarcomeres or fascicle lengths (Granzier et al., 1989; Hisey et al., 2009). However, *in vivo* studies showing greater FE during stretch in multi-joint contractions (Hahn et al., 2014). In contrast to that, Linnamo et al. (2006) reported the same FE at short and long muscle lengths during single joint contractions.

Due to the fact that the knee joint angle at T1 was different between the SSC and CON conditions—which led to a greater angle change in the CON conditions—we adjusted calculations of mechanical work to the actual knee angle range during shortening for better comparability of conditions. The results confirmed that the work performed during shortening is significantly greater for all SSCs compared to the corresponding CON contractions. Even absolute mechanical work was greater for all SSC contractions, although the knee angle change during shortening, and thus the absolute range, was greater in the CON conditions. In literature, several mechanisms are presented to explain an enhancement of mechanical work during shortening in a SSC. Activation dynamics, i.e., the time required to fully establish muscle activation, can play a major role in movements with or without a counter-movement (Fukutani et al., 2019b). However, muscle activation has no influence on our results of performance enhancement in SSCs, since we always had an isometric pre-activation phase before the dynamic phases. This means that for every level of intensity, trials for both conditions started with an identical muscle activity. Additionally, the stretch-reflex activity is discussed in order to contribute to increased work in SSCs. However, we consider the relevance of stretch-reflex contributions in our settings to be negligible or even non-existent for two reasons. First, stretch reflexes are expected in fast movements with fast muscle lengthening—such as hopping (Komi and Gollhofer, 1997)—and should not be relevant in our experiment with a movement velocity of 60°/s. Second, electrical stimulation was used in all submaximal trials, where clear SSC-effects were evident. In addition, by the visual inspection of the EMG data we did not identify any suspicious activation peaks at the onset of stretch. For these reasons, we are confident that the stretch-reflex also has no or negligible influence on our results.

As already mentioned at the beginning of this paper, this leaves two remaining mechanisms that most likely explain the entire SSC performance enhancement found in our work. The lengthening of passive elastic structures leads to the storage of elastic energy that is released during shortening and thereby contributes to enhanced work during shortening in the SSC (Finni et al., 2001). Additionally, tendon elongation can affect changes in muscle length, resulting in enhanced force-generating capability due to the force-velocity relationship (Hill, 1938; Ishikawa et al., 2005). The amount of tendon elongation is dependent on the force applied to the tendon (Reeves et al., 2003), which in our experiment means that with higher intensity, more energy can be stored in the tendon. This is true for the absolute forces and work during SSCs; however, the relative SSC-effect was nearly constant for all submaximal intensities ranging between 17 and 21% increased work, and slightly lower SSC-effects were found during MVCs. Contrary to what we expected, no influence of intensity on the relative mechanical work during shortening in the SSC was found, whereas the type of activation (electrically stimulated vs. voluntary) seems to have an influence on the SSC-effects.

The main question for this experiment was whether, and to what extent, stretch-induced force-enhancing effects within the contractile element of muscles can contribute to enhanced work during the shortening. In contrast to the discussed mechanisms above, a contribution of rFE-related mechanisms to increased work during shortening in SSC should be visible also after the shortening phase (Seiberl et al., 2015b). It was speculated that if rFE-related mechanisms contribute to the SSC-effects, then the increased force/torque should be triggered during the stretch phase, contribute to the performance enhancement during the shortening phase, and should be visible as a history-dependent property in the steady-state phases after the SSC. Joint torque in the steady-state (T3) after the pure CON trials was significantly depressed compared to the ISO reference contraction (range 6.6–12.7%, not significant for 20% intensity), which is in line with previous studies that reported a rFD between 5 and 25% at knee extensions (Lee et al., 1999, 2000; Altenburg et al., 2008; Dargeviciute et al., 2013). No statistical difference of rFD was found for pure CON muscle action and SSC at the same activation level, except for the test under maximal voluntary activation (CON_100%: rFD of 11.8% and SSC_100%: rFD of 3.1%), without any difference in m. vastus lateralis fascicle length and pennation angle. This indicates that mechanisms related to rFE are responsible for the less depressed steady-state torque in our experiment during the maximum voluntary SSC condition, which further means that the intensity of contraction and/or the type of activation in a SSC has an influence on this long-lasting component. Based on this finding, it might be concluded that there is an activation threshold where no significant rFD in the SSC of the m. quadriceps femoris remains. However, one needs to be careful comparing the submaximal and MVC conditions in this work, as there are important differences between voluntary and electrically elicited contractions. Electrically stimulated muscles do not mirror the asynchronous and varied firing frequencies voluntary activation shows (Lee et al., 1999) what likely influences muscle function as well. History-dependent effects show way more variability during voluntary contractions, than what we would expect from the ‘facts’ derived from animal models and electrically stimulated muscle action (Seiberl et al., 2015a). The reasons are still not well understood. We decided to use electrical stimulation for all submaximal contractions, since torque or EMG feedback-controlled trials are experimentally difficult to implement, and it can be only matched at the isometric state before and after the dynamic phase. This is not the case for MVCs, but extremely high stimulation intensities would be too painful and not tolerable for participants. For this reason, voluntary contractions were used for 100% intensity trials that additionally better represent everyday tasks or exercises like the squat, where we also have a stretch of the m. quadriceps femoris directly followed by a shortening contraction.

In literature, a current approach to explaining rFE is related to a titin-actin interaction (Rode et al., 2009; Herzog et al., 2016; Linke, 2018; Fukutani and Herzog, 2019). Titin stiffness increases with higher muscle activation, which could result in a reduced rFD in our SSC under maximal voluntary contraction. As previously reported, there are conflicting results in literature regarding the effects of rFE in the SSC (Groeber et al., 2019). Some authors reported that any stretch-induced rFE was abolished during the shortening in SSCs (Herzog and Leonard, 2000; Lee et al., 2001; Fukutani et al., 2019b), whereas others observed significantly higher force or torque values in the steady-state after the SSC compared to pure shortening muscle action (Seiberl et al., 2015b; Fortuna et al., 2017; Hahn and Riedel, 2018). Seiberl et al. (2015b) and Hahn and Riedel (2018) used electrical stimulation at 50–60% and 32.9% of MVC respectively, but they both had quite high shortening velocities in their test protocol, which is associated with reduced rFD (Herzog and Leonard, 2000). Additionally, Fortuna et al. (2017) reported that shortening affects rFD in SSC contractions in a time-dependent manner. The authors found that with a longer shortening time, a greater rFD was produced. However, the same authors still reported a reduced rFD after the SSC at an intensity level of 50–60% of MVC when the shortening phase was 1 s in length as was the case in our protocol. Their results come from the m. adductor pollicis, which—compared to the m. quadriceps femoris—is of a smaller size and has a short tendon. Since the mechanism for different rFD values with different muscle group sizes remain unknown (Chen et al., 2019), one can only speculate about the possible influence of muscle size on rFD in a SSC contraction.

## Conclusion

To our knowledge, this is the first study considering the steady-state torque after a SSC of the m. quadriceps femoris under maximal voluntary contractions and under different submaximal contraction intensities. We hypothesized that with increasing intensity, the SSC-effects are larger, possibly due to increased rFE.

In conclusion, we observed increased mechanical work during the shortening phase of the SSC for all contraction intensities. Additionally, reduced rFD in the SSC condition compared to CON was only found for 100% intensity. Under reduced activation, the stretch-induced force-enhancing effects were only visible during the shortening phase. Our results indicate that the magnitude of contribution of the potential mechanisms in SSCs of the m. quadriceps femoris changes with the intensity and type of activation. Furthermore, the complete attenuation of rFE in the lower intensities despite greater mechanical work during the shortening phase should be examined in future studies.

## Data Availability Statement

The raw data supporting the conclusions of this article will be made available by the authors, without undue reservation.

## Ethics Statement

The studies involving human participants were reviewed and approved by the Ethics Committee of the University of Vienna. The patients/participants provided their written informed consent to participate in this study.

## Author Contributions

MG, SS, and AB conceived and designed the experiment. MG performed the experiment. MG and SS analyzed the data. MG, SS, WS, and AB discussed the results, made a substantial contribution to the interpretation of data, and contributed to the elaboration of the manuscript. All authors contributed to the article and approved the submitted version.

## Conflict of Interest

The authors declare that the research was conducted in the absence of any commercial or financial relationships that could be construed as a potential conflict of interest.
